# Abstracts and Gleanings

**Published:** 1882-08-20

**Authors:** 


### ABSTRACTS AND GLEANINGS.

**Hot Water in Therapeutics.**—Dr. Douglas Morton, A. M., M. D., of Louisville, Ky., in Medical News, says:*

The use of hot water in the practice of gynecology has come to occupy so important a place as to make it hard for us to realize, looking back, that we could do without it; and although my own experience of its value in this department corresponds in the fullest degree with that of others, I yet wish to assert emphatically that in certain therapeutic applications its value appears to be even greater.

Several years ago I learned in my own personal experience that no agent relieves nausea and vomiting so satisfactorily and promptly as water as hot as can be drank. Since then I have used it in a large number of cases, and no remedy that I ever administered in any condition has proved more uniformly reliable. I have preserved records of many of these cases, but to transcribe them here would prolong this paper to a tedious length. I make, therefore, the following classification:

1. Cases in which nausea and vomiting occurred at the onset or during the course of acute febrile disease.

2. Cases in which these symptoms were caused by overloading the stomach when its functions had been impaired by protracted disease.

3. Cases in which they were produced by nauseous medicines (not emetics) at the time they were taken.

4. Cases of acute gastritis caused by the ingestion of irritants.

5. Cases in which these symptoms were purely reflex.

6. Cases of chronic gastritis.

7. Cases of colic in newly-born infants.

8. Cases of flatulent distention of the stomach in adults.

Class 1 contains a number of cases in which the value of hot water was most strikingly illustrated. Among them is a case of diphtheria and one of puerperal septicemia.

I might include also a case of tuberculosis in which the stress of the disease fell on the digestive apparatus. In each of these a half glass of hot water always gave prompt relief when every other remedy had failed.

Of all, however, the cases in which the use of this remedy seemed to produce the most impressive and the most permanently beneficial results were those of cholera infantum. In these it would often happen that hot water would not only itself be retained when absolutely everything else was rejected, but would immediately render the stomach tolerant of food. Taking advantage of this effect, my manner of using it is to give a few teaspoonfuls as often as it is necessary to administer food, and, immediate-

* Read before the Louisville, Ky., Medico-Chirurgical Society, Aug. 4, 1882.

ly afterward, while the stomach is fully under its sedative influence, to give food in small quantities. I have seen a number of children get well whose recovery I am confident was due solely to this treatment.

In class 3 there are cases in which the stomach had rejected all medicines for many hours together, but retained them readily when given in hot water as a vehicle.

In class 4, a victim of alcoholism, after a prolonged debauch, during which an enormous quantity of whisky had been drunk, had reached a point at which the stomach would no longer tolerate whisky or anything else. Hot water was given and retained, and the stomach rendered tolerant of food immediately.

In class 5 the patients were the subjects of vomiting in pregnancy. The effect produced in these cases was much less satisfactory than in others, yet sufficiently favorable to justify the positive statement that hot water is a remedy of considerable value.

Concerning classes 6, 7 and 8, which include cases of patients who were the subjects of various manifestations of indigestion, it suffices to make the general statement that the administration of hot water constituted a very important part of their management, and was followed almost invariably by good results. In the treatment of dyspepsia my rule is to order hot water in every case, to be taken before each meal, and as often at other times as suits the patient's convenience. I have found that this draught before meals causes a discharge of any undue amount of gas in the stomach by eructation. One of my dyspeptic patients told me that whenever he took food his stomach was distended by the gaseous products that came from the imperfect digestion of his last meal as to be incapable of getting the proper "grip" upon what he ate. This gentleman thinks that hot water did more toward curing him thany any of the many remedies he had tried. It affords relief in the same manner to young infants who suffer from colic, and I rarely have occasion to prescribe anything else for them.

In the case of another dyspeptic, who is the victim of gout also, attacks of indigestion were accompanied by very distressing palpitation of the heart. I saw this patient once during an attack which happened at night, and she was in a sad plight indeed. Her heart was beating with an irregularity of force and of rythm such as I had never seen before, and a horrible sense of impending dissolution made sleep impossible. I asked her to drink a large quantity of tepid water, hoping it would cause her to throw up the contents of her stomach. The water brought, however, was decidedly hot; but she drank it, and almost instantaneously the palpitation was relieved, and in a surprisingly short time, she passed into a tranquil sleep.

**The Bromides.**—The combinations of bromine occupy the prominent position which they do in modern medicine because of their influence over the nerve centres, whether through influencing their vascular supply or through some direct influence on the tissue itself, or through a combination of such action is not yet positively known. But through their *modus operandi* may not have been deffinitely determined, the fact nevertheless stands out that in the treatment of affections of the nervous system this class of remedies occupies a prominence not accorded any other drug in the pharmaopœia. It has largely supplanted opium in the treatment of nervous diseases, and is almost as indispensible in this direction as is quinine in the treatment of the effects of malaria.

In view of this extensive applicability it becomes important to know the methods, if there is a difference in methods, of its administration. That a variety of results may be secured through various methods of its exhibition scarcely admits of a doubt, and we have been much interested in an article on this division of the subject which appeared from the pen of Dr. Geo. M. Beard in the July, 1881, issue of the Journal of Mental and Nervous Diseases. The article calls attention to the fact that the bromides are by no means innocent agents or incapable of mischief, and that to secure their effects they must be intelligently prescribed and with circumspection. Much mischief has resulted from their routine administration:

Dr. Beard has submitted a number of propositions bearing on this question of the bromides, which are of much practical importance :

First. The object of using the bromides is usually to produce a deffinite effect—bromization, in a greater or lesser degree. Bromization acts therapeutically through being itself a disease, which operating on the tissues involved renders them less susceptible to our disturbing causes. It is of different degrees, varying from very mild sedation to profound stupor and even death, for the bromides may be pushed to the extinction of life:

Secondly. Differences in the degrees of individual susceptibility render it impossible to specify the quantity of the drug necessary to bromization. In those nervous affections in which it is necessary to induce a profound impression, as in epilepsy for instance, the remedy must be pushed until the effect is secured, and that regardless of the dose specified in the books.

Thirdly. When it is necessary to create a profound impression, care must be taken not to continue it too long. This rule does not apply with so much force in epolepsy and epileptiform affections. In any case it is not safe to give large doses unless the patient can be held sufficiently under observation to have the effects noted.

Fourthly. When the affections for which they are given render neccssary the continued use of the bromides, they should be combined with tonics. This has been long understood in the treatment of epilepsy, but it is not less necessary in other affections.

Fifthly. It is an advantage to combine a number of the bromides, and Dr. Beard gives preference to the following: Bromide of potassium, which contains 68 per cent. of bromine; bromide of calcium, containing 80 per cent.; bromide of sodium, containing 80 per cent.; bromide of ammonium, containing 81 per cent., and bromide of lithium, which contains 92 per cent. of bromine. Bromide of sodium has the advantage of being less liable to cause gastric irritation. The bromide of camphor, bromhydric acid, bromide of quinine, bromide of zinc and bromide of iron are also eligible combinations, and may, under certain circumstances, be substituted for those of the primary list.

Sixthly. There are persons who, although they may not have epilepsy or be subject to epileptoid conditions, find it necessary to continue in the use of the bromides to overcome or obviate attacks of nervous perturbation.

The condition of the circulation in the various parts affected must be considered in discussing the applicability of the bromides. It is the general conception that they act by creating an anæmia of the brain and cord, but Dr. Beard is of the opinion that they exert an influence sui generis. While they are not so markedly valuable in an anæmic condition they may nevertheless be given with benefit for a limited time in cases in which their employment would seem to be indicated regardless of the blood supply. In such cases, however, their use should not be long continued. They doubtless relieve passive congestions of the nerve centres, and through this same property it would seem that they should aggravate the anæmic condition.

There is a large field for the employment of the bromides in modern practice, and the propositions here given will assist the reader to an intelligent appreciation of their nature and the cases demanding their temporary and continued use.—*Ther. Gaz.*

**Koch at the German Congress for Internal Medicine.**— At the Congress for Internal Medicine, recently held at Weisbaden, Dr. Koch presented microscopic sections showing his tubercle bacillus. He also described his work, and announced again his conclusions.

The ensuing discussion was somewhat disappointing, owing, no doubt, to the fact that no one had time or opportunity to repeat Dr. Koch's experiments.

Drs. Aufrecht and Klebs accepted the demonstrations of Koch. The former even went so far as to assume that the centre of true miliary tubercles is filled, not with degenerated cells, but with micro-organisms, partly rod-bacteria, partly micrococci. Aufrecht had seen the organisms in tubercle like those found by Baumgarten as well as by Koch. Professor Klebs said that he had for some years occupied himself with the question of the infectiousness of tuberculosis, and he had tried to discover a disease-bearing organism. He was gratified at Koch's success, but warned him of the severe attacks his views would have to encounter.

Dr. Seitz asked some pertinent questions with regard to how Koch would explain, on his theory, the existence of hereditary phthisis, and of the frequent occurrence of phthisis in certain diseases, *e.g.*, diabetes.

Dr. Ruhle followed with a similar inquiry, and said that even if Koch's facts regarding inoculation of the tubercular bacilli were correct, the etiology of human phthisis would yet be unsolved.

Koch replied to these gentlemen, and, as his answer contains a very clear statement of his views, we give it quite fully.

It is, he said, a well-known fact that the development of micro-organisms is greatly influenced by the character of their nutritive media. Human bodies do not always offer equally good nurture ground for pathogenic bacteria. Some persons may, while most do not, inherit a system which is well calculated for the development of the tubercular bacilli. These former are the hereditarily disposed to phthisis. The distinction between phthisis and miliary tuberculosis must, said the speaker, fall to the ground. For such distinction only depends upon the mode and amount of bacilli infection. In acute miliary tuberculosis large numbers of the pathogenic organisms are poured into the blood. In phthisis, however, only one or a few get into the lungs. Thus in the lower animals, when a very few tubercular organisms are inoculated in the anterior chamber of the eye, there is a slow and perhaps local tuberculous infection. If large numbers, however, are inoculated, the animal soon dies of a general tubereulosis.

The above account includes the most important part of the debate, which afterward drifted off into a discussion regarding phthisis in children.

It will be seen that our German brethren take Dr. Koch's discovery calmly, and are not as yet inclined to consider it an epoch-making affair. By Dr. Koch's own explanation it is shown that the existence and infective power of a bacillus does not solve entirely the problem of the etiology of phthisis. This bacillus only grows upon suitable ground, *i. e.*, in the phthisically predisposed. We have yet to find exactly what constitutes or brings about this peculiar predisposition. Again, according to these experiments the inoculation of the bacillus always produces tuberculosis in lower animals. Must we infer, therefore, that lower animals are always predisposed? We shall be very glad to find that Koch's conclusions are justified by further studies and experiments. But the evidence at present calls for much caution in interpreting their significance.—*N. Y. Med. Record.*

**New Method of Inducing Sleep.**—A French writer, according to the Cincinnati Lancet, says that if the eyelids be closed and opened as fast as possible twenty or thirty times, an irrisistible inclination to sleep will follow in a few seconds. The plan is best adapted to insomnia from nerve troubles. We have tried it with some advantage, but not so much as the statement implies. The point to be gained by men of busy heads in order to induce sleep, is to divert the current of cerebral action away from all exciting and disturbing thought. This may be done by any one of a thousand expedients which have been contrived, to occupy the attention by some trivial exercise of memory or imagination which will crowd out the perturbing themes and substitute others neither exciting nor disagreeable. Such are the counting of an imaginary flock of sheep jumping one by one over a fence; counting the vibrations of an imaginary pendulum; counting your own respirations, giving one or two to each inspiration and each expiration; counting a hundred backwards; repeating the alphabet backwards; calling to mind all the names of persons beginning with the several letters of the alphabet, etc. This plan may be utilized by applying it to geography, history, biography, botany, and other branches of knowledge; recalling as far as possible all names of rivers, or cities, or lakes beginning with A, then B, and so forth; or the names of distinguished men in like manner, or of plants. A medical student or a physician might trace the arteries or nerves, or recount the bones of the skeleton, if he desire a very dry and sleepy subject. All these exercises wear out in time and must be changed and newly contrived ones substituted, for there is no limit to their range. Mental recitations of poetry or prose may be resorted to. There are also mechanical methods of overcoming insomnia, particularly if the "fidgets" be present. Such are getting out of bed and applying the flesh brush freely; friction with a coarse and wet towel. Franklin used to take what he called an air bath, stripping himself completely and walking back and forth over the floor briskly. Some mechanical methods act indirectly on the brain, as the opening and closing of the eyes above mentioned. Dr. Hartshorne's method of inducing anæsthesia by rapid breathing may furnish some hints in this direction. Most persons have felt the effect on the head of blowing at a fire with the mouth and lungs—what dizziness and approach to unconsciousness it induces. Similar movements favor sleep. We have often induced sleep by a few exhalations and inhalations, both as complete as possible. It is singular how long the lungs will remain quiescent after these forced movements, before the call for oxygen rene .vs the normal action of the respiratory muscles.—*Pacific Med. Jour.*

**Vesico Vaginal Fistula.**—Dr. Muscroft ha recently devised and performed a new operation for closing the vulva in incurable cases of vesico-vaginal fiistula. The patient upon whom the operation was performed was a primipara twenty-three years of age, who had been delivered of a dead child after a sever labor of three days' duration. The result had been a vesico-vaginal fistula, and separation of the urethra from the bladder. The vagina was much contracted, and the buttocks and thighs were excoriated by the action of the urine. General treatment and bougies were tried for some time, and after the lapse of nearly a year from her entrance into the hospital, where she came under the author's notice, an unsuccessful attempt was made to attach the remnant of the urethra to the bladder, which was followed by further sloughing of the urethra. About four months later, as her condition was unimproved, the author decided to close the vulva, leaving an orifice for the escape of the urine at its posterior commissure. The anæsthetic used was chloroform. The mucous membrane was removed from the labia majora, the clitoris and the nymphæ were excised, and the opening was closed with five sutures of polished annealed steel wire. The thighs were bound together, and a solution of sulphate of iron, of the strength of half a drachm to a pint of water, was applied externally by compresses, and injected into the vaginal and pelvic cavity. The sutures, with the exception of the one nearest the outlet, were allowed to ulcerate away. The urine escaped constantly for a time, but by degrees the opening contracted, and the patient began to have control of the urinary discharge. When she was discharged from the hospital she had retentive power for two hours at a time. The clitoris was removed because the operation was thought to be *neater* by this procedure, and more likely to succeed, and because it might have a tendency, to repress venereal desire. The patient's general health was very good when she was allowed to go home.

[The paper of which the foregoing is an abstract, was read before the Cincinnati Academy of Medicine, and called forth some deservedly adverse criticism. It is a step backward to resort to mutilations like this, in view of the careful and successful operations of Emmet, Bozeman, and others, in the worst possible cases of fistula.]—*N. Y. Med. Journal.*

**How to Count a Rapid Pulse.**—Dr. Abbott, in N.Y. Medical Record, says: I notice an item referring to a statement by Prof. Pribram, that "he had a case of 'vagus neurosis' in which the pulse reached three hundred beats per minute." In the context is an inference that such a pulse cannot be counted. In 1870, while experimenting upon the action of alcohol on birds, I found myself unable to count the rate of the heart's action by the usual method when the contractions were over two hundred and forty per minute (the heart's action is very distinct in birds). By the following simple method I was able to count to two hundred and eighty without difficulty: During a definite part of a minute, one-fourth usually, with a common lead pencil, dots were made upon a sheet of paper synchronous with the heart-beats, as heard over the cardiac region. The dots were then counted, and the number calculated for the whole minute. A pulse of four hundred could be taken in this way, provided each pulsation were distinct enough to be discriminated by the touch. The indistinctness of the separate pulsations of the heart alone fixes the limit to the use of this method, as the human hand is capable of making intelligently and with accuracy, at the rate of four hundred and fifty dots per minute, for thirty seconds, which rate is probably beyond not only that of the human heart, but also of the pulse of any of the lower animals available for experiment. I have had a sufficient experience with this method to know that it is of practical value, especially with children.

I have been astonished at the actual rapidity of the heart's action, in a few cases which I should have considered as beyond counting by any other method. All who have used the sphygmograph know how difficult it is to get a tracing when the heart is beating to one hundred and forty even; so that this instrument, to say nothing of the fact of its expense and inconvenience, is not nearly so well adapted to the taking of a very high pulse as even the simple finger and watch method. By the method described above, all movements, whether of the body or not, that can be seen, felt or heard, can be counted up to four or five hundred per minute, provided they are sufficiently distinct to be discriminated. Fortunately, the appliances are always at hand, and their use so simple, and at the same time so accurate, that it need never be said that a pulse is "too rapid" for counting.

**Sunstroke.**—The New York City Board of health has issued the following circular on the prevention of sunstroke, which we reprint for the benefit of our readers:

Sunstroke is caused by excessive heat, and especially if the weather is "muggy." It is more apt to occur on the second, third or fourth day of a heated term than on the first. Loss of sleep, worry, excitement, close sleeping-rooms, debility, abuse of stimulants, predispose to it. It is more apt to attack those working in the sun, and especially between the hours of eleven o'clock in the morning and four o'clock in the afternoon. On hot days wear thin clothing. Have as cool sleeping-rooms as possible. Avoid loss of sleep and all unnecessary fatigue. If working indoors and where there is artificial heat (laundries, etc.), see that the room is well ventilated.

If working in the sun, wear a light hat (not black, as it absorbs the heat), straw, etc., and put inside of it, on the head, a wet cloth or a large green leaf; frequently lift the hat from the head, and see that the cloth is wet. Do not check perspiration, but drink what water you need to keep it up, as perspiration prevents the body from being overheated. Have, whenever possible, an additional shade, as a thin umbrella when walking, a canvass or board cover while working in the sun. When much fatigued, do not go to work, but be excused from work, especially after eleven o'clock in the morning on very hot days, if the work is in the sun. If a feeling of fatigue, dizziness, headache or exhaustion occurs, cease work immediately, lie down in a shady and cool place, apply cold cloths to and pour cold water over head and neck. If any one is overcome by the heat, send immediately for the nearest good physician. While waiting for the physician, give the person cool drinks of water or cold black tea or cold coffee, if able to swallow. If the skin is hot and dry, sponge with or pour cold water over the body and limbs, and apply to the head pounded ice wrapped in a towel or other cloth. If there is no ice at hand, keep a cold cloth on the head, and pour cold water on it, as well as on the body.

If the person is pale, very faint, and pulse feeble, let him inhale ammonia for a few seconds, or give him a teaspoonful of aromatic spirits of ammonia in two tablespoonfuls of water with a little sugar.—*Jour. Chem.*

**Where is a Man's Stomach?**—One would suppose that by this time the position of the human stomach should have been definitely settled by physiologists, but according to Dr. Leshaft, Professor of Anatomy at St. Petersburg, the statements on this point in the text-books are erroneous. The Lancet gives the following summary of his conclusions, based on careful observations upon more than twelve hundred bodies:

The stomach does not, as is usually asserted, lie horizontally in the abdominal cavity, but vertically, so that the fundus touches the diaphragm; the smaller curvature and pylorus are to the right, and the larger curvature is to the left. Its position is in the left hypochondrium, and the situation of the pylorus is in the vertical line formed by a continuation of the right margin of the sternum. If the stomach is enlarged, no one part can be alone displaced, but all parts are equally moved by the distention. The arrangement of the muscular fibres of the stomach is such that food entering it is moved toward the pylorus, where it can be most thoroughly mixed with the gastric juices, and in them passes back along the centre of the cavity to the fundus, where the resistance is least. This movement of the food along the wall to the pylorus, and back again along the centre is rendered possible by the form of the organ, and it is probable that it is to this movement that the peculiar shape of the fundus is due. As is well known, the fundus is absent in newly-born children. Thus the shape of the stomach determines the long retention of food in the organ for the purposes of digestion, and its slow passage through the pylorus. If the transverse colon is distended with gas, it may rise to the left of the stomach as high as the fourth intercostal space, and even as high as the fourth rib. If the coils of the small intestine are similarly distended, the lower part of the stomach may be pressed forward, and the stomach may assume a more oblique position. Even a large stomach, accustomed to dietetic repletion, maintains a vertical position, but the pylorus is moved a little upward and to the right.—*Jour. Chem.*

**Elixir Iodo.**—Dr. O. Millard, of Flint, Michigan, says: Under the name of "Elixir Iodo," said to be a chemical compound and patented in July, 1882, we were struck with the utter inconsistency of the specification and claim of the patent, and also disgusted with the loose way in which that portion of the patent office at Washington pertaining to chemistry is run. Henry A. Tilden, in his specification states that the iodide and bromide produced, are combined with chloride of calcium, and is based upon certain reciprocal chemical equivalents; and further on, in the same specification, he states that he does not limit himself to the precise proportion of the iodide, bromide or chloride.

If the compound was based upon the known laws of chemistry and upon certain reciprocal chemical equivalents, it would be impossible to change their proportions *ad libitum.*

The real fact of the matter is that Mr. Tilden exposes his ignorance of chemical law in the specification, which in itself is prima facie evidence that no chemical compound of the kind has been discovered, but in its stead a mixture, such as any physician might chance to make in a single prescription at any time. The gentleman states that he has invented a new and useful "chemical compound." Isn't that cheek? *I invented!*

The first man who made a barrel of common salt (chloride of sodium) by evoporation must have invented the same chemical mixture if he made the salt at Saginaw.

The "Iodo" is not a chemical compound any farther than the individual salts are concerned; taken as a whole it is a *mixture* of chemicals. Iodide and bromide of calcium, iodide and bromide of magnesium do not unite chemically with chloride of calcium, and the amount of cheek necessary to make the claim staggers us.—*Therapeutic Gazette.*

**Digestive Wine.**—Professor Schmitt, Lille, offers to supply a formula for a digestive wine, equal, if not superior, to the most vaunted products (L'Union Pharm., November, 1880). He recommends pharmacists to make their own pepsin. He advises that the rennet solution, after the preliminary treatment of the Codex, should be treated with a mixture of sulphate and phosphate of soda instead of hydro-sulphuric acid, as those salts, in excess even, are rather likely to be of advantage than otherwise. This solution should be evaporated in a sand-bath to the consistence of a firm extract. In this he incorporates 10 per cent of the purest glycerine. This will keep soft, and this he calls *pepsin extractive.*

Next he takes malt and crushes in a linseed mill or in a marble mortar, macerates it for 24 hours with ten times its weight of cold water, and afterwards presses through linen. Strong alcohol is to be added to the liquid until 45° is marked on the centesimal alcoholmeter. The liquid becomes turbid, and yields a considerable precipitate. After standing for another 24 hours the liquid is filtered, and alcohol is again added until 66° is marked. After standing another 24 hours the liquid is to be carefully decanted, and may be distilled for the recovery of the alcohol. The muddy precipitate deposited is to be evaporated to a firm consistence, 10 per cent. of glycerine is to be added, and a maltine extractive is obtained.

For the preparation of a wine of pepsin and diastase of maltine, take—

| | Grammes. |
|---|---|
| Pepsine extractive | 5 50 |
| Maltine extractive | 5 50 |
| Common salt | 5 00 |
| Good brandy | 45 00 |
| Old Chablis wine | 400 00 |
| Grenache wine | 500 00 |

Each teaspoonful of this wine would contain about 20 centigrammes of digestive ferments.—*Monthly Review.*

**Two Hundred and Fifty Cases of Malaria Treated with the Tincture of Iodine.**—Dr. Robert B. Morison, in Maryland Medical Journal, says: The use of iodine in the treatment of intermittent fever is by no means new.

Stille and Maisch, in their dispensatory, under the head of iodine and its uses, say: "In intermittent fever iodine displays decidedly curative virtues, both in tropical malarial regions and in those of temperate zones. The tincture has been given in doses of from five to fifteen minims largely diluted."

So successful have we been with iodine we always order it now in intermittent fever of the acute sort. We give it to pregnant or nursing women; we give it where there is diarrhœa or constipation, and we have only heard, out of these 250 cases, from 2 where the chills have not been controlled by it. The dose is a pleasant one, and the opinion of the patients is decidedly in favor of taking it instead of the bitter malarial mixture. In only one case was nausea caused by it. In this case the dose was decreased to one-half the regular dose and a cure effected. We had no case of iodism, nor did we discover any albuminuria. The patients, as is natural after an acute disease, generally need a tonic, and this we always order in the form of iron or one of the bitters.

In conclusion, I will say, the treatment is an established fact at the dispensary, and is carried out by the experience of others elsewhere and in private practice. Dr. Hoffman has tried it at the jail with success and is, after having seen it so often given, quite as much convinced as I am of its efficacy.

**Lutidine as an Antidote for Strychnia.**—Messrs. Greville Williams and Waters have discovered (British Medical Journal) an antidote for strychnia in the organic base, first prepared by the former, by distilling cinchona with caustic potash, and to which he assigned the name *beta* lutidine. Having ascertained, by experiments upon frogs, that *beta* lutidine causes a distinct increase in the tonicity of both cardiac and voluntary muscular tissues; also retardation of the heart's beat; that it arrests the inhibitory power of the vagus; and that by its action upon the nerve-cells of the spinal cord. it, in the first place, lengthens the time of reflex action, and then arrests that function; they proceeded to test its direct counter-action to strychnia. The brains of frogs were destroyed in the usual way. An animal was then treated with *beta* lutidine till reflex action disappeared, when the subsequent administration of strychnia was not followed by the usual results. To another frog strychnia was given till strychnia tetanus was produced, when it was found that the subsequent administration of lutidine caused the tetanus to pass off. The almost simultaneous administration of the two bases was not followed by tetanus. It is not unlikely that a substance so powerful will have some positive therapeutical value.—*N. Y. Med. Record.*

**Criteria of Insanity.**—One of the pupils of Esquirol asked his teacher to furnish him with a sure criterion for distinguishing the limit that separates reason from insanity. The next day Esquirol invited to dinner his pupil and two individuals, one of whom was most correct in his appeaıance and in his language, while the other was very loquacious, full of himself, and of his future. When taking leave the pupil reminded his master of the criterion which he asked of him on the previous evening. "Answer the question for yourself," said Esquirol. "You have just taken dinner with a madman and a sane individual." "Oh," answered the pupil, "the problem is not difficult; the sane man was that distinguished and well-informed man; as to the other, he was a chatterer and a fool who ought really to be shut up." "Ah," replied

Esquirol, "you are making a great mistake; the one you took to be so very wise, believes himself to be God the Father, and affects in his manners the reserve and dignity which he believes belong to his position; he is a patient at Charenton. As to the young man whom you took to be a fool, in him you see one of the most illustrious of French authoos—he is M. Honore de Balzac.—*British Med. Jour.*

**Bacilli in Tubercle.**—*Lance:* On May 8th, Messrs. Watson, Cheyne & Nelson, in the Pathological Laboratory at King's College, demonstrated the bacilli in tubercle which have recently caused so much excitement in medical circles. Dr. Goltdammer—Dr. Koch's private assistant—has brought over to England several specimens of bacilli prepared by Dr. Koch, and these were submitted for the first time to the inspection and criticism of English pathologists. In addition to bacilli in tubercle, those of leprosy, of septicæmia, and of erysiplatous inflammation, the bacillus anthracis was shown. Among the pathologists present were Mr. Lister, Drs. Wilks, Payne, Pye-Smith, Beale, etc., and altogether about seventy gentlemen minutely examined the specimens. There can be no doubt whatever as to the presence of the organisms in tubercle-formations and in the diseases referred to, although their exact significance may still be questioned. Dr. Goltdammer also showed a test-tube in which the tubercle-virus was being cultivated in blood-serum. The same specimens were also exhibited at the soiree of the Royal Society on the 10th inst., and attracted a large amount of attention from the biologists as well as from the physicians and surgeons who were present.—*Mich. Med. News.*

**Calabar Bean in Obstinate Constipation.**—We notice a report of the efficacy of calabar bean in the Berlin Klin. Wochenschrift which illustrates the process of reasoning based on a knowledge of the physiological action of drugs. It is known that calabar bean produces tetanus of the intestinal muscular coats in animals, and hence will bring about the forcible expulsion of the contents of the intestines. Dr. Shæter has on this ground employed it in obstinate constipation depending on atony of the muscular coats, such as is often observed in women, and in old men. The results justified his expectations, for severe cases have yielded in less than 24 hours after administration of the drug. He employs a solution of the following formula: Extracti physostigmatis, 0.05 grm.; glycerini, 10.00 grms. Six drops every three hours during the day. The editor would suggest the combination of this agent with cascara sagrada in those cases where that drug does not produce sufficient peristaltic action. Such a combination might prove of advantage by securing in addition to the action of the calabar bean the peculiar stimulating action on the secretory organs connected with the alimentary canal, which has made so much deserved reputation for the bark of the rhamnus purshiana. —*Ther. Gaz.*

**Agaricus in the Treatment of Night-Sweating.**—Dr. Wolfenden finds that atropia yields excellent results when given in doses of 1-70 of a grain. It is, however, a dangerous drug to use, on account of its poisonous properties. Dr. Wolfenden therefore prefers to employ agaricus, which is of equal value to atropia, while it is quite harmless, since ten grains too much or too little produce no toxic effects. Agaricus is a light, bulky, brown powder, of very bitter taste, and is best administered in the form of confection, with a little jam. Twenty grains are usually quite sufficient given at bed time, though thirty grains may be necessary to check the sweating completely, the only inconvenience attending the administration of large doses being the great quantity of the powder. Patients, however, make no objection to the bitter taste, etc., when they find how much benefit they receive from its use. Dr. Wolfenden has administered it in nearly forty cases of phithisis with complete success. The only ill effects which have been noticed are, first sickness, which stops on elimination of the dose; secondly, diarrhœa, which can be averted by combination with one or two grains of Dover's powder.—*Glasgow, Md., Journal.*

**Congress of German Surgeons.**—Among the subjects discussed by this Congress during its meeting in Berlin last June were the treatment of wounds and the possibility of finding a dressing which shall possess both disinfectant and absorbent properties. Charcoal, sand, and "glass wool" steeped in sulimate, paper ashes, and dried peat were among the materials recommended for this purpose. One surgeon was of the opinion that the iodoform era in surgery was over, the general impression being that iodoform does not meet the demands of a model dressing.

For the radical cure of hernia the use of alcohol as an injection near the sac was advised in place of the oak-bark solution employed in America.

A case of spleen extirpation was reported by Crede, Jr., and the use of the esophagoscope and gastroscope was demonstrated by Mikulicz.

There was an exhibition of excised stomachs, and reports were read on excision of the thyroid, the transplantation of muscle, and the resection of the lung.

The characteristic feature of nearly every paper read was something new in pathology or therapeutics or operative procedure.—*Ex.*

**The Effect of Thapsia Garganica on the Skin.**—Two cases in which an eruption on the face was produced by the application to the chest of a plaster made from the root of thapsia garganica have been recorded by Comby. These plasters are a popular remedy in France. A local irritant effect is produced in a few hours, and the next day myriads of small vesicles and pustules are produced at the spot and in its vicinity, the skin between them being bright red. In one of the cases described there was also, when the plaster was removed, swelling of the face, which rapidly increased to such a degree as to close the eyes, and on the reddened skin vesicles and bullæ appeared. There was no fever or enlargement of the glands, and the eruption gradually subsided. In the other cases two plasters had been applied to the chest, and a very similar eruption appeared on the face, which ran a similar course. The eruption appeared simultaneously in all the parts affected, and did not spread as does erysipelas.—*Lancet.*

**The Influence of Certain Remedies upon the Milk-Secretion.**—As the result of a clinical and experimental investigation, Dr. Max Stumpf, of Munich, gives (Deut. Arch. fur Klin. Med.) the following as his observations of the effect of certain remedies upon the secretion of human milk:

I.—Alterations in quantity of the milk:

1. Iodide of potassium causes a considerable decrease in the total quantity of milk.

2. Alcohol, morphia, and lead do not alter the quantity secreted.

3. Salicylic acid appears to increase slightly the flow of milk.

4. Pilocarpin is not a remedy furthering the milk-secretion.

II.—Alterations in the quality:

1. Potassium iodide disturbs the glandular functions so much as to lead to uncertainty as to its qualitative effects.

2. Alcohol and alcoholic drinks increase only the fatty constituents of the milk. As dietetic agents for the purpose of increasing the milk are therefore to be discarded.

3. Lead, morphia and pilocarpin scarcely, if at all, affect the quality of the milk.

4. Salicylic acid appears to increase the sugar.

III.—Discharge of poisons in the milk:

1. Iodine appears quickly in the milk, and in man rapidly disappears after the discontinuance of its administration, but in the herbivora it is more persistent. As regards the proportion of the iodine discharged in this way, it bears no constant relationship to the dose taken, and varies in different individuals. "The therapeutic application of iodized milk is therefore out of the question." The drug is discharged not in the form of alkaline salt, but in some combination with casein.

2. In the herbivora alcohol does not pass over into the milk.

3. Lead appears only in traces, but remains for several days after the ingestion of the remedy has ceased.

4. Salicylic acid, when given in large doses, appears also in very slight quantity in the milk, in man rather more than in the lower animals —*Med. Times.*

**Jaborandi.**—Dr. Ryder says: "I believe jaborandi to possess the power of eliminating from the human system almost any specific poison, by means of the skin, if resorted to at once and before the poison has had time to set up its peculiar action."

**Sensible.**—"There is no greater blunder than to object to a journal on account of its large advertising department. The size of this department is the key to a journal's success and the index of its prosperity." It was a wise man who wrote these words.—*Exchange.*

